# Researches on Mathematical Relationship of Five Elements of Containing Notes and Fibonacci Sequence Modulo 5

**DOI:** 10.1155/2015/189357

**Published:** 2015-10-01

**Authors:** Zhaoxue Chen

**Affiliations:** School of Medical Instrument & Food Engineering, University of Shanghai for Science and Technology, Shanghai 200093, China

## Abstract

Considering the five periods and six qi's theory in TCM almost shares a common basis of stem-branch system with the five elements of containing notes, studying the principle or mathematical structure behind the five elements of containing notes can surely bring a novel view for the five periods and six qi's researches. By analyzing typical mathematical rules included in He tu, Luo shu, and stem-branch theory in TCM as well as the Fibonacci sequence especially widely existent in the biological world, novel researches are performed on mathematical relationship between the five elements of containing notes and the Fibonacci sequence modulo 5. Enlightened by elementary Yin or Yang number grouping principle of He tu, Luo shu, the 12534 and 31542 key number series of Fibonacci sequence modulo 5 are obtained. And three new arrangements about the five elements of containing notes are then introduced, which have shown close relationship with the two obtained key subsequences of the Fibonacci sequence modulo 5. The novel discovery is quite helpful to recover the scientific secret of the five periods and six qi's theory in TCM as well as that of whole traditional Chinese culture system, but more data is needed to elucidate the TCM theory further.

## 1. Introduction

Combining the 10 celestial stems and 12 earth branches, the Chinese sexagenary cycle can be formed, which is widely used in Chinese ancient culture as the most important means of counting time of years, months, or dates. In TCM (Traditional Chinese Medicine), given that each year is associated with a different combination of stems and branches over a cycle of sixty years, all possible climatic constellations of the year may sequentially occur in a specific way, which can be characterized by the stem-branch combination closely related with the five periods and six qi. In other words, the doctrine of the five periods and six qi explains relationships ancient Chinese observers assumed to exist between climate and a broad range of natural phenomena, including human health and illness. And the concepts of the five periods and of the six qi were introduced to distinguish among and specify climatic characteristics of well-defined time periods. By drawing on notions of a cyclical recurrence of calendric terms and by adopting the doctrines of yin yang and of the five elements, an attempt was made to order what may at first glance appear to be disorder, namely, the occurrence of rain and wind, dryness, cold, and heat in the course of the four seasons and over the years. Knowledge of a distinct regularity uncovered in frequent climatic changes not only permitted an understanding of the generation, growth, maturity, and death of numerous phenomena in nature in general but also, more importantly, enabled man to integrate himself into eternal laws governing all existence. The doctrine of the five periods and six qi is outlined in the Su wen in seven “comprehensive discourses,” which comprises about one-third of the entire text of the Su wen. But the origin of the notions is unclear and no parallel literary sources outside the Su wen are known that could be used to date the early development of these thoughts [1]. All the same, there are also some important clues in other fields of ancient Chinese culture which can be used as a reference for researchers of the five periods and six qi in TCM. Therein, theory of the five elements of containing notes originated at least before Qin Dynasty is the most notable one, where each combination of a stem and a branch is also vital for description of yin yang and five elemental characteristics and each attributed to a corresponding element named as the so-called five elements of containing notes [2, 3]. The na yin wu xing, that is, the element representing the stem-branch of one's birth-year, is often used to judge one's fate by folk fate calculators up to nowadays and in the Book of the Master Who Embraces Simplicity by Ge Hong (who is a famous Taoist priest in Jin Dynasty) it is recorded that the fate corresponding to the five elements of containing notes decided by the stem-branch combination corresponding to one's birth year can be used as a guidance of color selecting of the medicine to be taken; Ge Hong says in volume number 11 of his inner book of the Master Who Embraces Simplicity: “…According to the book of Yu ce ji and the book of Kai ming jing,…, if one's fate is soil, he is not fit to take medicine with cyan color; medicines with red color are not fit for persons of metal fate; white color is not fit for wood fate; yellow color is not fit for water fate and black color is not fit for fire fate. That is exactly because, according to meaning of the five elements, the wood restricts the soil, the soil restricts the water, the water restricts the fire, the fire restricts the metal and the metal restrict the wood…”.  Although some of Ge Hong's ideas are contentious with a mysterious tendency of a common Taoist, it is true that his Handbook of Prescriptions for Emergencies inspired the modern discovery of artemisinin [4]. No matter in theory of the TCM five periods and six qi or in theory of the five elements of containing notes, the same 60 cyclical stem-branch combinations by years are paid much attention and related judgments or doctrines are formed based on the stem and branch related theory of yin yang and five elements. Considering the five periods and six qi's theory of TCM almost shares a common basis of stem-branch system [1, 2], studying the principle or mathematical structure behind the five elements of containing notes can surely bring a novel view for the five periods and six qi's related researches in TCM.

The Fibonacci sequence, that is, 1, 1, 2, 3, 5, 8, 13, 21, 34, 55, 89, 144, 233,…, is a famous series universally used in various modern disciplines such as computer science, optimizing theory, biological mathematics and physics, number theory and combinatorics, and material science [5–9]. Even there is a special formal periodical (namely, The Fibonacci Quarterly) dedicated to Fibonacci sequence related topics and researches [10]. In particular, having close relationship with the Golden Section Number, it is an interesting mathematical sequence that widely exists in the biological world. For example, there is a peculiar pattern in the flower petals of nearly all the flowers; the number of their petals is one number of the Fibonacci sequence. Small flowers of a sunflower, the heart of chrysanthemum, squama on surface of pinecones, and tumors-like structure of pineapple all have shown similar two near-perfect spirals in two opposite spiral directions, respectively. And the ratio of two spiral numbers has close relationship with the Fibonacci sequence. In the pinecones the ratio is 5 : 8, the pineapple is 8 : 13, Marguerite daisy is 21 : 34, and the sunflower is 34 : 55,…; the series of number couples are all from two adjacent numbers of the Fibonacci series exactly. Any face plate of the Asteraceae family has the same characteristics with the sunflower. In animal cells, hollow cores of microtubules constituted by protein polymer, which form the cell cytoskeleton, help to maintain a certain shape and act as “nervous system” of cells. Typical mammalian cell microtubule is constituted by 13 original fibers, of which 5 are dextrorotation fibers and 8 are laevorotation ones (herein, 5, 8, and 13 are all adjacent Fibonacci numbers). Moreover, people have occasionally found a double-microtubule with an outer layer, and it is constituted by 21 original fibers, which happens to be the next number in Fibonacci series. Moreover, it is well known that molecules of the B-DNA expose a double-helix geometric structure, and the helix length of the double-helix DNA structure is 34 angstroms and its radius is 21 angstroms. 34 and 21 happen to be the two adjacent numbers in the Fibonacci sequence [6].

Moreover, the Fibonacci sequence is also found to have close relationship with traditional Chinese culture. Elizabeth Moran and her coauthors have discovered that four of feng shui's (feng shui is another mysterious system in traditional Chinese culture system with almost the same theory basis as TCM) fundamental principles correspond to numbers in the Fibonacci sequence: Taiji (1), yin and yang (2), heaven, earth, and human qi (3), five phases (5), and eight trigrams (8) [11]. And according to some other researchers, the Fibonacci numbers also have close relationship with the most famous two key diagrams of He tu and Luo shu in ancient Chinese culture [12, 13].

In this paper, according to some typical mathematical rules included in He tu, Luo shu in TCM, novel associations are discovered between the five elements of containing notes and the Fibonacci modular sequence, which can set up a mathematical bridge between the five elements of containing notes widely existent in Chinese traditional culture and abundant modern Fibonacci series related researches. The novel discovery is of great value to reduce the mysterious sense of ancient Chinese culture and is quite helpful to uncover the final scientific secret of the five periods and the six qi's theory in TCM.

## 2. Materials and Methods

### 2.1. Obtaining of Two Key Subsequences of Fibonacci Sequence Modulo 5

**Figure 1:**
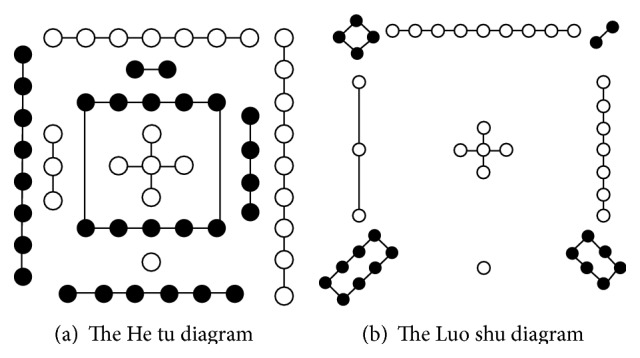
The He tu and Luo shu diagram.

The He tu and Luo shu are two of the most famous diagrams in Chinese traditional culture as well as in TCM system. As shown in Figure 1 [1, 11], according to legend, both He tu and Luo shu initially emerged as groups of black and white dots, where each group of black dots has even dot number and the other groups composed of white dots have odd number of dots, respectively. In particular, either He tu or Luo shu has shown the same proneness to emphasize group number of 5 and the group of dots with number 5 is exactly positioned in the center of He tu and Luo shu which are arranged as the shape of a cross. Further according to the Hong fan section in Shang shu, number one is water; number two is fire; number three is wood; number four is metal; number five is soil [1]. The number of different groups of dots in He tu or Luo shu can thus form a direct association with the five elements' theory in TCM or the five elements of containing notes.

The Fibonacci sequence has a recycled property while performing modular operation by an integer *n*. While setting the value of *n* be 5, that is, the center number of He tu or Luo shu, the period is equal to 20 and the corresponding recycled sequence can be listed as below [14]:

 1 1 2 3 0 3 3 1 4 0 4 4 3 2 0 2 2 4 1 0

Suppose each index value of numbers 1, 2, 3, 4, 5, 6, 7, 8, 9, and 10 in He tu or Luo shu is equal to each number itself; then no matter He tu and Luo shu or 10 stems and 12 branches in TCM can all be partitioned into two groups of Yin and Yang based on the parity of index values. In other words, for 10 stems and 12 branches, those with odd index numbers are attributed to the Yang group and the others with even numbers are grouped as Yin, which just corresponds to the white or black dots group in He tu and Luo shu, respectively. Similarly, according to parities of the index of each number the obtained 20 Fibonacci recycled sequence numbers by modulo 5 above can also be divided into two groups: subsequence Yang and subsequence Yin. The grouping result is listed as below:

 subsequence Yang: 1 2 0 3 4 4 3 0 2 1,
 subsequence Yin: (1 3) 3 1 0 4 2 2 4 0 1 3,

for analyzing convenience; all the numbers in the two subsequences are rearranged into two 5-tuples and all numbers in subsequence Yin are circularly right-shifted by 2 positions. Apparently the obtained subsequences of Yang and Yin are just composed of series of 1 2 0 3 4 and 3 1 0 4 2 together with their corresponding palindrome, that is, two subsequences both taking 1 2 0 3 4 and 3 1 0 4 2 as the key sequence, respectively. In mathematics, the number 0 is just equal to 5 in the viewpoint of operation modulo 5; therefore, the key number series of 1 2 0 3 4 and 3 1 0 4 2 can also be seen as 1 2 5 3 4 and 3 1 5 4 2.

### 2.2. New Discovery on Arrangement of the Five Elements of Containing Notes

Based on traditional Chinese culture, each of the containing notes associates a unique symbol as well as an attribute and Hong fan number of five elements with a stem-branch combination and two neighbored stem-branch combination pairs share the same symbol and attribute [2, 3]. The naming of the associated symbols, deriving, and relationship between such association and ancient Chinese music theory are beyond the scope of this paper; those who are interested in it can refer to related literatures directly. All stem-branch combinations and each corresponding symbol as well as the associated attribute and Hong fan number of corresponding five elements are all summarized as in Table 1 based on description in [2, 3].

Actually all the stem-branch combinations are just the permutation and combination between Yang group of stems and branches and Yin group of them. And the thorough building process of associations shown in Table 1 is still a mystery. While studying the rules behind the stem-branch combinations, the author found a new arrangement of the stem-branch combinations based on a simple and determinate transforming operation from the original arranging order in traditional Chinese culture which is demonstrated in Table 1 by the “number” column. The discovery of this new arrangement can help to provide research clues of mathematical structure of the five elements of containing notes. That is, take the JiaXu YiHai pair (11th and 12th stem-branch combinations in Table 1) as the initial pair and take equation (*m* + 22) mod 60 (herein “mod” represents modular operation) as a recursive rule to obtain the successive one of all recycled pairs; for example, if substituted with *m* = 11 and 12, the index of the second recycled pair can be obtained; we have

 (11 + 22) mod 60 = 33
 (12 + 22) mod 60 = 34;

then look up the indexes 33 and 34 in Table 1; the second pair BingShen DingYou can be searched out. Similarly, the index of the third pair can be computed as (33 + 22) mod 60 = 55, (34 + 22) mod 60 = 56,…, and finally, the whole periodical new arrangement can all be obtained. And the 5 by 6 array form of the new arrangement is shown in Table 2.

For comparison convenience, substitute each stem-branch pair in the new arrangement shown in Table 2 by its corresponding Hong fan number according to Table 1; it produces a 5 by 6 number array:

**Table:** 

2	2	2	4	4	4
5	5	5	1	1	1
3	3	3	2	2	2
4	4	4	5	5	5
1	1	1	3	3	3

Looking up from up to bottom, it is obvious that each column of the number array is one of the recycle-shifting sequence of 12534 just by one position in the left or right direction, that is, 25341 or 41253, and looking from the end there are exactly three appositions of 31542 31542.

Above discovery is not only a special case. Stem-branch combinations and their corresponding Hong fan number given in Table 1 can also derive two other arrangements as listed in Tables 3 and 4 based on the 12 branches or base number 8 after extending one duplicate of whole 60 elements.

Obviously, in each column of Table 3 the Hong fan numbers are the recycle-shifting sequence of 12534 by a certain number. And in each column of Table 4, by recycling view, the Hong fan numbers all show a similar appositional pattern of three same recycle-shifting sequences of 31542.

## 3. Results, Discussions, and Conclusions

In this paper, enlightened by elementary Yin or Yang number grouping principle of He tu, Luo shu, and stem-branch theory, the 12534 and 31542 key number series of Fibonacci sequence modulo 5 (the center number of He tu or Luo shu) are obtained. And by simple and determinate transforming operation of original order of the stem-branch combinations widely existent in traditional Chinese culture, a new 5 by 6 arrangement about the five elements of containing notes is firstly introduced, which has shown close relationship with the two obtained key subsequences of Fibonacci sequence modulo 5. As the new derived arrangement is directly acquired from the original indices of stem-branch combinations by fixed mathematical operation, the rule implied in the new arrangement also reflects that of the original arrangement of stems and branches in traditional Chinese culture. Another two derived arrangements about the five elements of containing notes also show close relationship with sequence 12534 or 31542 respectively; therefore it can be deduced that the five elements of containing notes must have underlying profound mathematical relationship with the Fibonacci modular sequence. Actually, besides applications in fate judgments or in ancient Taoist system, the five elements of containing notes associated with 60 stem-branch combinations have also close relationship with traditional Chinese musical system [3]. And for ancient Chinese people, it is very common and natural to describe the musical and calendric laws in the same form based on stems and branches. In their opinions, the climate throughout a whole year is closely related with certain musical notes denoted by a stem-branch combination corresponding to the year as the five periods and six qi in TCM do. Moreover, the Fibonacci sequence widely exists in various modern disciplines especially in the biological world, and as a special traditional medicine system, herbs from the biological world are widely used in TCM. Therefore, although researches in this paper are mainly performed based on arrangements of the five elements of containing notes, considering it almost shares a common basis of stem-branch system with the theory of the five periods and six qi in TCM, the mathematical relationship of five elements of containing notes and Fibonacci sequence modulo 5 found in this paper is undoubtedly quite helpful to recover the scientific secret of the five periods and six qi's theory in TCM as well as that of whole traditional Chinese culture system, but more data is still needed to elucidate the TCM related theory in future researches.

## Figures and Tables

**Table 1:** Associated stem-branch combinations, symbols, and the attributes and Hong fan numbers of five elements.

Sequence number	Stem-branch combination	Symbol	Attribute and Hong fan number
1	JiaZi		Metal
2	YiChou	The metal in the sea	4

3	BingYin		Fire
4	DingMao	The fire in the stove	2

5	WuChen		Wood
6	JiSi	The wood of a great forest	3

7	GengWu		Soil
8	XinWei	The wayside soil	5

9	RenShen		Metal
10	GuiYou	The metal on the sword-blade	4

11	JiaXu		Fire
12	YiHai	The fire on the hill-top	2

13	BingZi		Water
14	DingChou	The brook water	1

15	WuYin		Soil
16	JiMao	The soil on the city-wall	5

17	GengChen		Metal
18	XinSi	The metal on the white candle	4

19	RenWu		Wood
20	GuiWei	The willow wood	3

21	JiaShen		Water
22	YiYou	The spring water in a well	1

23	BingXu		Soil
24	DingHai	The soil on the roof of a house	5

25	WuZi		Fire
26	JiChou	the thundering fire	2

27	GengYin		Wood
28	XinMao	The wood of the pine or cypress	3

29	RenChen		Water
30	GuiSi	The flowing water	1

31	JiaWu		Metal
32	YiWei	The metal in the sand	4

33	BingShen		Fire
34	DingYou	The fire at the foot of a hill	2

35	WuXu		Wood
36	JiHai	The wood on a plain	3

37	GengZi		Soil
38	XinChou	The soil on the roof of a house	5

39	RenYin		Metal
40	GuiMao	The metal on the paper money	4

41	JiaChen		Fire
42	YiSi	The fire of a lamp under cover	2

43	BingWu		Water
44	DingWei	The water of the heavenly river	1

45	WuShen		Soil
46	JiYou	The soil of the highway station	5

47	GengXu		Metal
48	XinHai	The gold of the hairpin	4

49	RenZi		Wood
50	GuiChou	The wood of the mulberry tree	3

51	JiaYin		Water
52	YiMao	The water of a great stream	1

53	BingChen		Soil
54	DingSi	The soil in the sands	5

55	WuWu		Fire
56	JiWei	The heavenly fire	2

57	GengShen		Wood
58	XinYou	The wood of the pomegranate	3

59	RenXu		Water
60	GuiHai	The water of the sea	1

**Table 2:** The 5 by 6 array form of the new arrangement.

JiaXu YiHai	BingShen DingYou	WuWu JiWei	GengChen XinSi	RenYin GuiMao	JiaZi YiChou
BingXu DingHai	WuShen JiYou	GengWu XinWei	RenChen GuiSi	JiaYin YiMao	BingZi DingChou
WuXu JiHai	GengShen XinYou	RenWu GuiWei	JiaChen YiSi	BingYin DingMao	WuZi JiChou
GengXu XinHai	RenShen GuiYou	JiaWu YiWei	BingChen DingSi	WuYin JiMao	GengZi XinChou
RenXu GuiHai	JiaShen YiYou	BingWu DingWei	WuChen JiSi	GengYin XinMao	RenZi GuiChou

**Table 3:** New arrangement based on the 12 branches.

JiaZi YiChou4	BingYin DingMao2	WuChen JiSi3	GengWu XinWei5	RenShen GuiYou4	JiaXu YiHai2
BingZi DingChou1	WuYin JiMao5	GengChen XinSi4	RenWu GuiWei3	JiaShen YiYou1	BingXu DingHai5
WuZi JiChou2	GengYin XinMao3	RenChen GuiSi1	JiaWu YiWei4	BingShen DingYou2	WuXu JiHai3
GengZi XinChou5	RenYin GuiMao4	JiaChen YiSi2	BingWu DingWei1	WuShen JiYou5	GengXu XinHai4
RenZi GuiChou3	JiaYin YiMao1	BingChen DingSi5	WuWu JiWei2	GengShen XinYou3	RenXu GuiHai1

**Table 4:** New arrangement based on base number 8 after extending one duplicate of 60 elements.

Jiazi YiChou4	BingYin DingMao2	WuChen JiSi3	GengWu XinWei5
RenShen GuiYou4	Jiaxu YiHai2	BingZi DingChou1	WuYin JiMao5
GengChen XinSi4	RenWu GuiWei3	JiaShen YiYou1	BingXu DingHai5
WuZi JiChou2	GengYin XinMao3	RenChen GuiSi1	JiaWu YiWei4
BingShen DingYou2	WuXu JiHai3	GengZi XinChou5	RenYin GuiMao4
JiaChen YiSi2	BingWu DingWei1	WuShen JiYou5	GengXu XinHai4
RenZi GuiChou3	JiaYin YiMao1	BingChen DingSi5	WuWu JiWei2
GengShen XinYou3	RenXu GuiHai1	JiaZi YiChou4	BingYin DingMao2
WuChen JiSi3	GengWu XinWei5	RenShen GuiYou4	JiaXu YiHai2
BingZi DingChou1	WuYin JiMao5	GengChen XinSi4	RenWu GuiWei3
JiaShen YiYou1	BingXu DingHai5	WuZi JiChou2	GengYin XinMao3
RenChen GuiSi1	JiaWu YiWei4	BingShen DingYou2	WuXu JiHai3
GengZi XinChou5	RenYin GuiMao4	JiaChen YiSi2	BingWu DingWei1
WuShen JiYou5	GengXu XinHai4	RenZi GuiChou3	JiaYin YiMao1
BingChen DingSi5	WuWu JiWei2	GengShen XinYou3	RenXu GuiHai1
